# Drawing the mind: assessing cognitive decline through self-figure drawings

**DOI:** 10.3389/fpsyg.2025.1558675

**Published:** 2025-05-07

**Authors:** Limor Goldner, Amit Pery, Johanna Czamanski-Cohen, Alex Nisara Jaroenkajornkij, Aviel Ben-Bassat, Gefen Avraham, Bussakorn Binson, Rachel Lev-Wiesel

**Affiliations:** ^1^University of Haifa, Haifa, Israel; ^2^Chulalongkorn University, Bangkok, Thailand

**Keywords:** self-drawing, older adults, cognitive functioning, Alzheimer’s disease dementia, mild cognitive impairment

## Abstract

**Background:**

Drawing requires the integration of visual perception, spatial processing, motor planning, and executive functions, but few studies have explored the potential connection between drawings, cognitive decline and dementia.

**Aim:**

This study compared self-figure drawings of elderly individuals with Alzheimer’s disease (AD) and mild cognitive impairment (MCI) to those with normative cognitive functioning.

**Method:**

A total of 496 older adults from Thailand and Israel (*M*age = 73.97, 70% women) participated in this study. Participants completed the Montreal Cognitive Assessment (MoCA-5) and then engaged in a self-figure drawing task. The drawings were categorized into eight groups based on their graphic characteristics. MANCOVA was used to examine differences between the drawing groups, t-tests were used to examine cultural differences, and Chi-square tests were used to examine differences and associations between the drawing groups and the MoCA-5 scores or categories.

**Results:**

We found that normative cognitive performance was associated with adapted portraits, whereas moderate to severe impairment correlated with schematic, disorganized, and unusual portraits. Cultural differences were also observed: the Thai participants had higher MoCA-5 scores than their Israeli counterparts and fewer differences in drawing group distribution.

**Conclusion:**

These findings suggest that self-figure drawings may reflect the cognitive status of older adults, with more detailed and adapted drawings indicating better cognitive functioning.

**Implications for practice:**

Self-figure drawings can be used as a complementary tool for assessing cognitive decline in diverse populations. However, cultural differences in drawing styles and cognitive test performance underscore the need for culturally sensitive approaches to dementia assessment and research.

## Introduction

Alzheimer’s disease dementia (AD), the most common form of dementia in the Western world, is defined by cognitive decline. This decline is documented through standardized testing in two or more domains, such as difficulty remembering recent events, language problems, disorientation, mood swings, loss of motivation, and self-neglect, all of which interfere with daily functions. AD symptoms impact individuals, their families, and society (Lane et al., 2018). Globally, it is estimated that 50 million individuals currently have dementia, with 10 million new cases each year (World Health Organization, 2019). This number is estimated to triple by 2050, primarily due to the anticipated increase in aging populations in Asian Pacific countries (Bai et al., 2022). In contrast to Israel, which is considered a relatively young society with a population growth rate of around 1.9% (Weinreb and Shraberman, 2021), Thailand is transitioning into an aging society, with individuals aged 60 and above comprising 16.7% of the total population, amounting to approximately 11 million people. According to the Thai National Health Examination Survey, the prevalence of dementia among older adults in Thailand is 8.1% (Thaipisuttikul et al., 2022).

Cognitive functioning in Alzheimer’s disease declines gradually over time. In the early phases, there is only slight cognitive impairment. By contrast, individuals in the advanced stages of Alzheimer’s lose their capacity to engage and respond to their environment (Castellani et al., 2010). The prevalence of AD or a related form of dementia is expected to increase to 115.4 M in 2050 (Alzheimer’s Association, 2023).

The Alzheimer’s Disease Continuum is a conceptual framework that characterizes the stages of AD and the underlying pathophysiological changes throughout the disease. The AD continuum is often divided into several stages based on the severity of cognitive symptoms and underlying pathology (Alzheimer’s Association, 2023; Breijyeh and Karaman, 2020). The earliest stage in the continuum is preclinical, which refers to the period before any cognitive symptoms become apparent. During this stage, individuals may exhibit signs of biomarker changes in the brain, including amyloid positron emission tomography (PET), cerebrospinal fluid (CSF) concentrations of amyloid and tau proteins, and plasma concentrations of amyloid, but these changes are often not detectable by standard clinical tests, and individuals may appear asymptomatic (Marasco, 2020; Molinuevo et al., 2012; Snyder et al., 2014).

As the disease progresses, individuals may develop Mild Cognitive Impairment (MCI). In MCI, individuals experience mild cognitive decline, such as memory loss and difficulties with problem-solving. Then, individuals enter the Mild Dementia stage, where cognitive decline becomes more severe and interferes with daily activities (Bradfield, 2023; Lim et al., 2022; Wiels et al., 2021), thus necessitating assistance with basic tasks such as dressing and bathing (Brodaty and Donkin, 2009; Phan et al., 2019; Kiselica and Alzheimer’s Disease Neuroimaging Initiative, 2021).

Finally, in the most severe stage, known as advanced dementia, individuals experience complete cognitive function loss that requires around-the-clock care. At this stage, individuals may experience difficulty swallowing, weight loss, and loss of bladder and bowel control (Powell, 2018; Ready and Ott, 2003). Mentally, studies indicate higher rates of late-life depression and a reduction in perceived dignity in the early stages of dementia (Klůzová Kráčmarová et al., 2022; Ismail et al., 2017; Panza et al., 2010). These findings underscore the need to recognize the early indicators of dementia throughout the Alzheimer’s disease continuum.

### Drawings, self-figure drawing, and dementia

The use of drawings to assess cognitive development is well-established (Mazzotta et al., 2015; Stafstrom et al., 2002). Drawing is considered a complex activity requiring the activation of cognitive, visuospatial, and motor abilities. Psychological research has consistently confirmed the effectiveness of using drawings to assess cognitive development and cognitive abilities (Goodenough, 1926; Flanagan and Motta, 2007; Wilson and Ratekin, 1990). This effectiveness can be attributed to the demands of drawing, which require the involvement of cognitive, visuospatial, and motor skills (Abell et al., 1994; Imafuku and Seto, 2022; Mazzotta et al., 2015). Thus, drawing may be a valuable tool for assessing the early signs and progression of dementia (Ericsson, 1996; Ericsson et al., 2001; Stanzani Maserati et al., 2018). Individuals with dementia often experience difficulties with planning, motor control, and visuospatial coordination (Paganini-Hill and Clark, 2007). These changes are likely to impact their ability to accurately depict their surroundings and symbols, as well as incorporate creative ideas in their drawings that tap into key cognitive abilities, such as perception, symbolism, and fine motor skills (Crutch and Rossor, 2006).

One widely used projective technique is the Draw-A-Person test (DAP), also known as the Human Figure Drawing (HFD) test. Developed by Goodenough in 1926, it originally served as an assessment tool to evaluate cognitive functioning in children, measuring their intellectual maturity and cognitive development. Goodenough instructed the children to draw a person using only a paper and pencil. The drawings were scored based on the inclusion of body parts, proportions, details, and realism. Two decades later, Harris (1963) demonstrated that DAP test scores were correlated with IQ scores on the Stanford-Binet test in children aged 3–15, thereby making the DAP an inexpensive cognitive screening tool (Goodenough, 1926; Wilson and Ratekin, 1990).

More recent comprehensive approaches have employed a greater number of indicators and the aggregation of signs to differentiate between normative and pathological populations of children (Naglieri, 1988; Goldner et al., 2021). A modified version of the DAP was developed by Lev-Wiesel (1999). This version explicitly prompts the person drawing to reflect on themselves directly. The participants are instructed to draw a picture of themselves in pencil.

The onset of degenerative disease is frequently accompanied by distortions in self-identity and symptoms of depression, which may be sufficient to account for observed changes in artistic style (Bonoti et al., 2015; Crutch and Rossor, 2006; Jungwirth et al., 2009).

These changes can impact individuals’ ability to accurately depict their surroundings, symbols, and creative ideas in their drawings. Studies have shown that the HFDs of AD patients exhibit specific characteristics, including smaller, shorter, and less detailed figures positioned away from the center of the sheet, with omitted sensory organs such as the eyes, mouth, and nose.

For example, Ericsson (1996) compared the HFD characteristics of 86 demented individuals aged 75 and above to those of 361 non-demented individuals in terms of body details and structural characteristics. There were fewer body details in the figures, the figures were shorter, and there was a tendency to place the figure in the upper left corner of the paper in the demented group. More recently, Lev-Wiesel and Hirshenzon-Segev (2003) examined the extent to which Alzheimer’s disease was reflected in the self-figure drawings of 32 individuals aged 62–87. The findings revealed an association between the severity of cognitive decline and more childlike stages of artistic development. Additionally, the body and eyes were often omitted in most of the drawings.

Stanzani Maserati, et al. (2018) examined the differences in HFD in 112 AD patients, 100 mild cognitively impaired patients, and 104 healthy controls. The AD patients drew smaller figures with fewer details than the MCI patients or the controls. The human figures drawn by the MCI patients had an intermediate body height compared to those drawn by AD patients and the healthy controls. The head-to-body ratio in the figures drawn by the AD patients was larger than that of both the control group and MCI patients. However, the relative size of the human figures to the page was notably smaller in AD patients.

These studies, although insightful, have several shortcomings. For example, Lev-Wiesel and Hirshenzon-Segev’s (2003) study examined a small Israeli sample. The Stanzani Maserati, et al. (2018) article failed to provide crucial details on sample size, thus limiting the generalizability of their findings. Additionally, these studies did not examine cultural differences. The current study aims to address these shortcomings by exploring how self-figure drawings differ among older adults with Alzheimer’s disease (AD), those with mild cognitive impairment (MCI), and healthy controls, as well as how self-figure drawings of elderly Israeli and Thai individuals vary according to different degrees of dementia severity. We compared Western society to a non-Western society known for its aging population to highlight these differences. We hypothesized that individuals with memory decline would exhibit distorted and simplistic schematic drawings, while those with normal cognitive functioning would produce complete, detailed, and realistic drawings. Given the lack of findings on cultural differences, we did not establish a specific hypothesis regarding cultural variations.

## Methods

To obtain our aims, we conducted a correlative study that examined the relationship between HFD and Cognitive functioning in two different representative cultural samples.

### Participants and procedure

Four hundred and ninety-six individuals over the age of 60 participated in this study (*M*age = 73.97, *SD* = 9.26, 70.2% women, *n* = 347). To avoid ethical violations, we only approached participants with mild AD who were willing to participate in the study and could provide informed consent as evidenced by their caregivers’ reports. Of these, 77% were Israeli, and the remainder were Thai. Half of the participants were married (49.9%), 35.3% were widowers or widows, and the remainder were divorced (14.8%). A quarter of the participants (25.3%) lived alone in their own homes, 67.6% lived with a partner in their own homes, and the remainder (7.1%) lived in a nursing home. Over half of the sample (54.5%) had a junior high or high school education, 22.0% held a technical diploma, and 23.5% had a college degree.

After obtaining ethical approval from the Ethics Committee of the Faculty of Welfare and Health Sciences at the University of Haifa (approval # 387/21), research assistants (trained MA students in the healthcare professions) recruited participants through Facebook-targeted pages for the elderly, nursing homes, and day centers for the elderly. They contacted potential participants and explained the objective of the study and procedures. They also clarified that participants could withdraw from the study without repercussions. Upon signing the informed consent form, a 30-min data collection session was conducted in a comfortable and safe environment. During these sessions, the participants completed the MoCa-5 to evaluate their cognitive functioning and the self-drawing task and provided demographic information.

### Measures

#### Self-figure drawing

The participants were asked to draw themselves on a sheet of A4 paper with a pencil without using an eraser, with no time limit, as stipulated in the instructions for the DAP-SPED (Lev-Wiesel and Hirshenson-Segev, 2003). No further instructions were given. Any questions regarding the drawing were answered with “as you wish.”

Prior studies indicated that individuals experiencing cognitive decline, especially those with MCI and AD, are at a higher risk of developing depression and a depleted sense of self, which can further worsen cognitive deficits (Ismail et al., 2017; Jungwirth et al., 2009). Therefore, we asked participants to draw a representation of themselves rather than producing a generic figure. A self-portrait allows for a more personal and introspective expression, potentially revealing subtle aspects of identity, emotional state, and cognitive function that might not emerge through a standard figure drawing.

##### Coding of the drawings

The drawings were coded based on their characteristics. Inspired by the works of Lev-Wiesel and Hirshenson-Segev (2003) and Stanzani Maserati et al. (2018), the authors—two certified art therapists and two art therapy students—classified the drawings into groups using a global approach. This classification process considered the organization, overall impression, and aggregation of visual elements, addressing both content and style. Content analysis focuses on the inclusion of specific elements such as a human figure, portrait, or other object. Style analysis examined elements such as figure size and placement, emphasis or omission of body parts, line quality and integration of the contour, shading, completeness, and level of detail, developmental stage of the drawing, and the degree of distortion or realism in the representation of objects. All four authors collaboratively coded the drawings, resolving any disagreements through consensus. This analysis resulted in the identification of eight distinct drawing categories.

**Figure 1:**
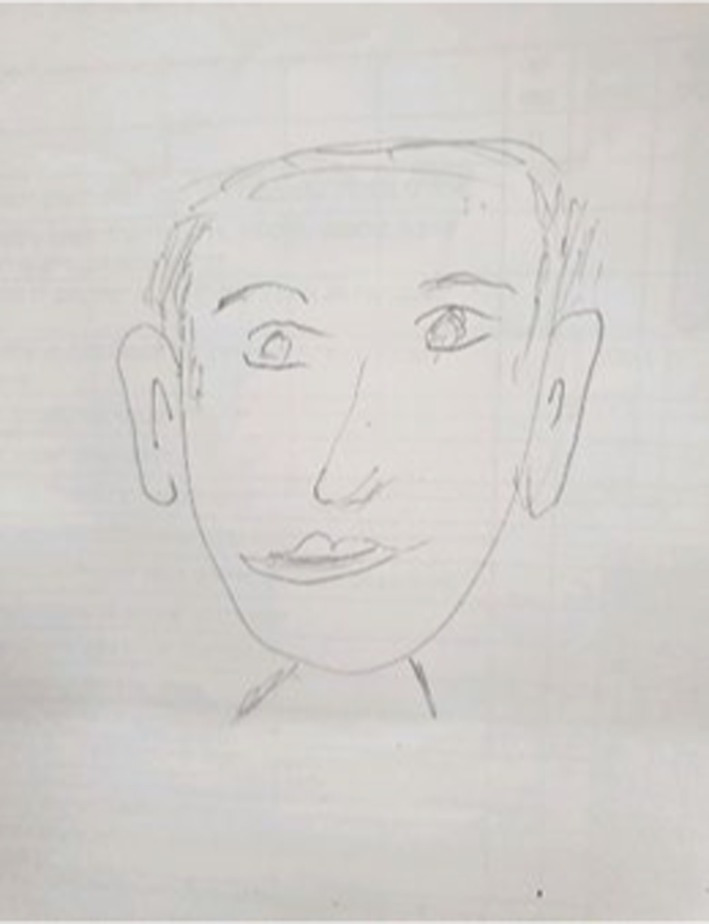
An example of adapted face drawing.

The first group (*n* = 57, see Figure 1), labeled ‘Adapted Face Portraits,’ was composed of drawings that only included the upper body and/or face. These drawings depicted all the facial features in a realistic manner, with a relatively complete outline. The drawings had a comparable adaptive appearance and reflected emotional investment in the drawings.

**Figure 2:**
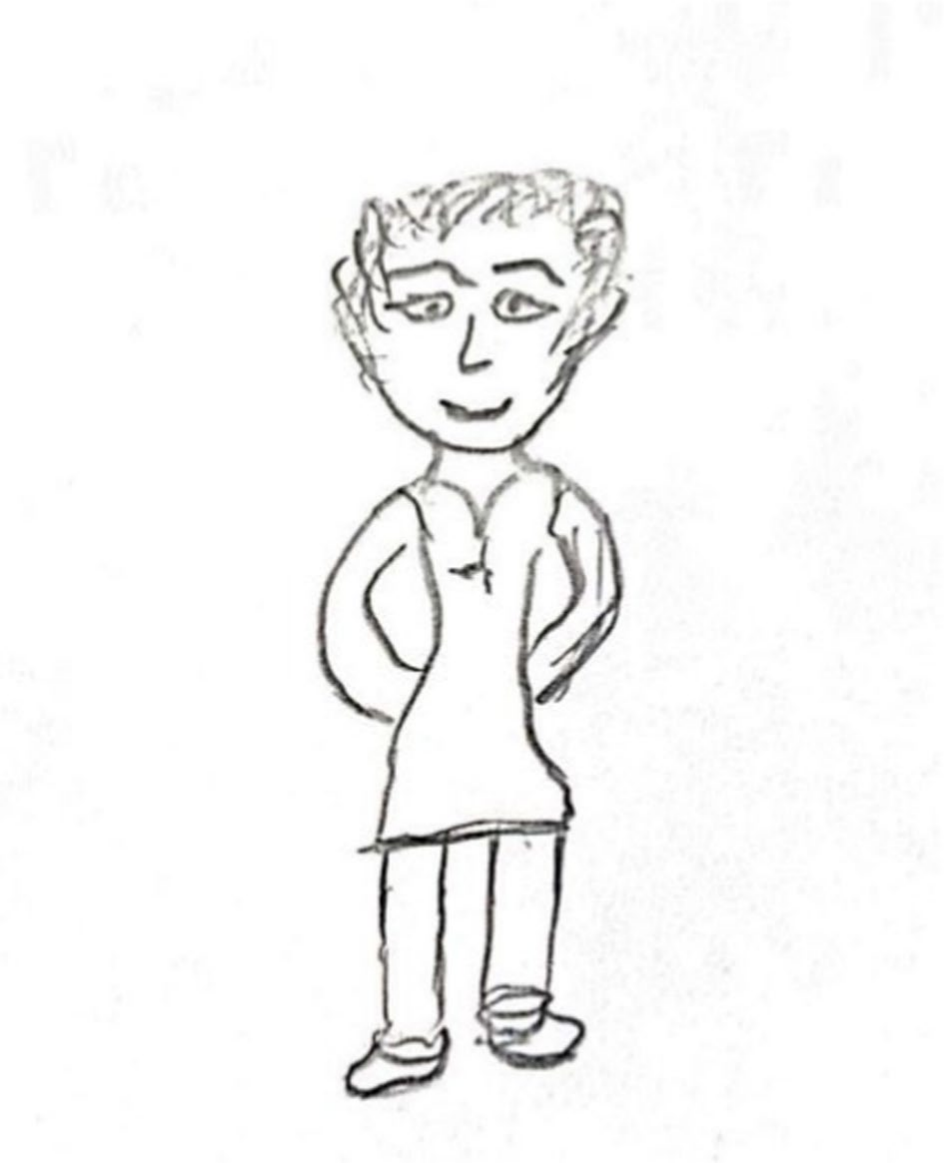
An example of adapted full body drawing.

The second group (*n* = 70, see Figure 2), labeled ‘Adapted Full Body Portraits,’ was composed of drawings of complete or nearly complete figures with age-appropriate appearances and minimal distortions.

**Figure 3:**
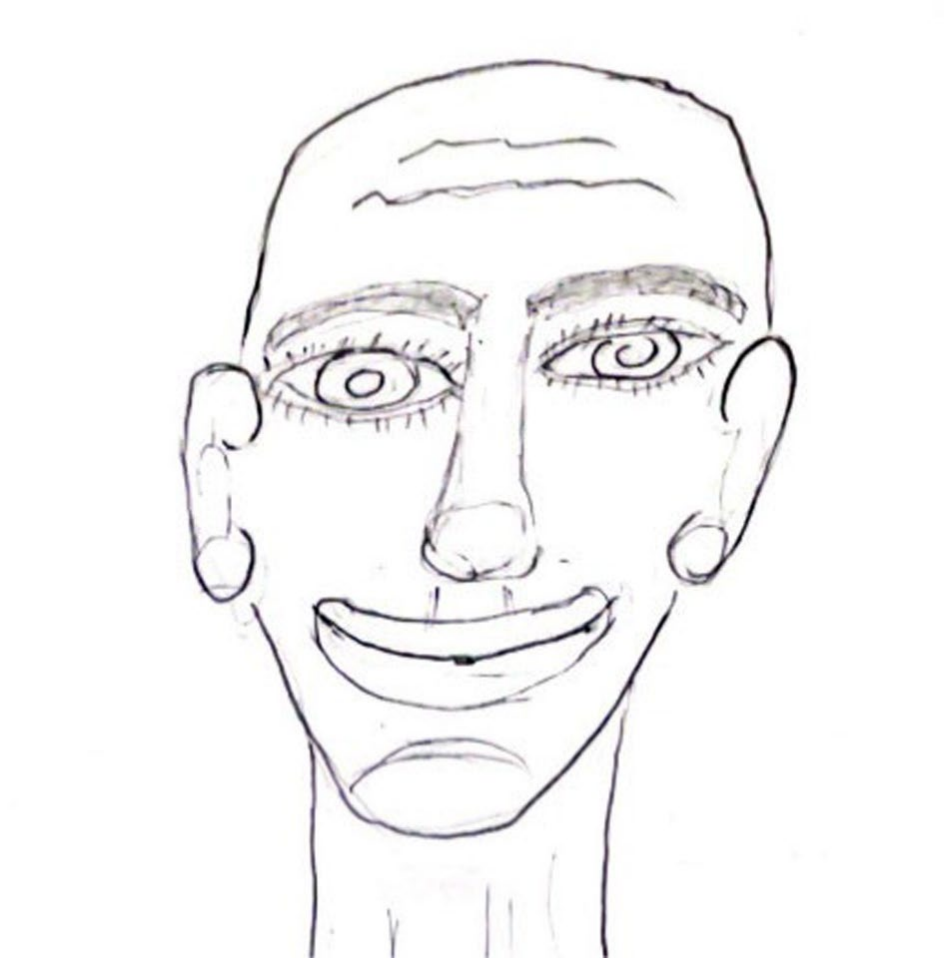
An example of exaggerated face drawing.

The third group (*n* = 34, see Figure 3), labeled ‘Exaggerated Face Portraits,’ included drawings with the upper body and/or face, depicting all the facial features exaggeratedly, sometimes with an incomplete outline and excessive focus on the head and sensory organs.

**Figure 4:**
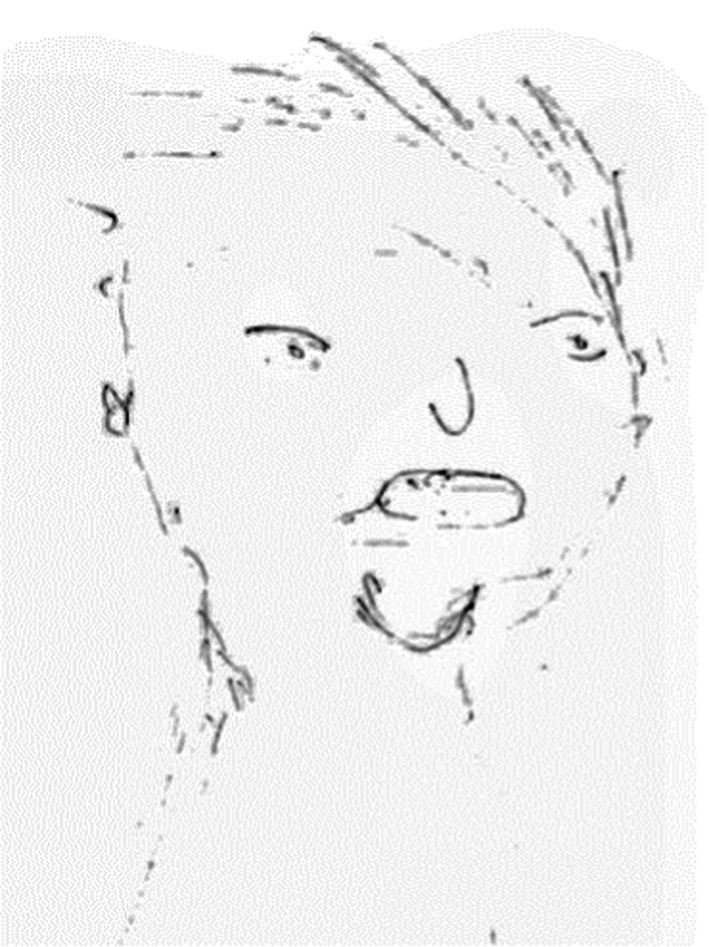
An example of disjointed face and body drawing.

The fourth group (*n* = 57, see Figure 4), labeled ‘Disjointed Face and Body Portraits,’ was composed of drawings characterized by a full or partially complete body with a poor contour, missing outlines, or emphasized body boundaries. The figures sometimes lacked hands or feet. These drawings conveyed a sense of discontinuity or effort in depicting the figure, sometimes with disproportions.

**Figure 5:**
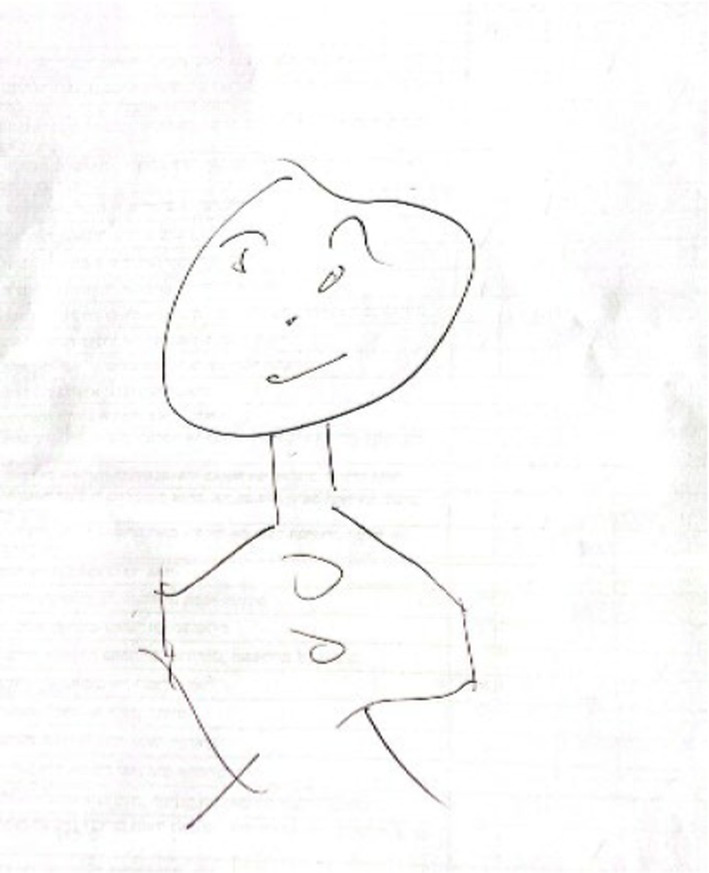
An example of schematic body drawing.

The fifth group (*n* = 50, see Figure 5), labeled ‘Schematic Body Portraits,’ was composed of figures characterized by a schematic body with a sketchy, less developed appearance, lacking individuality, presenting a generic look, with missing details.

**Figure 6:**
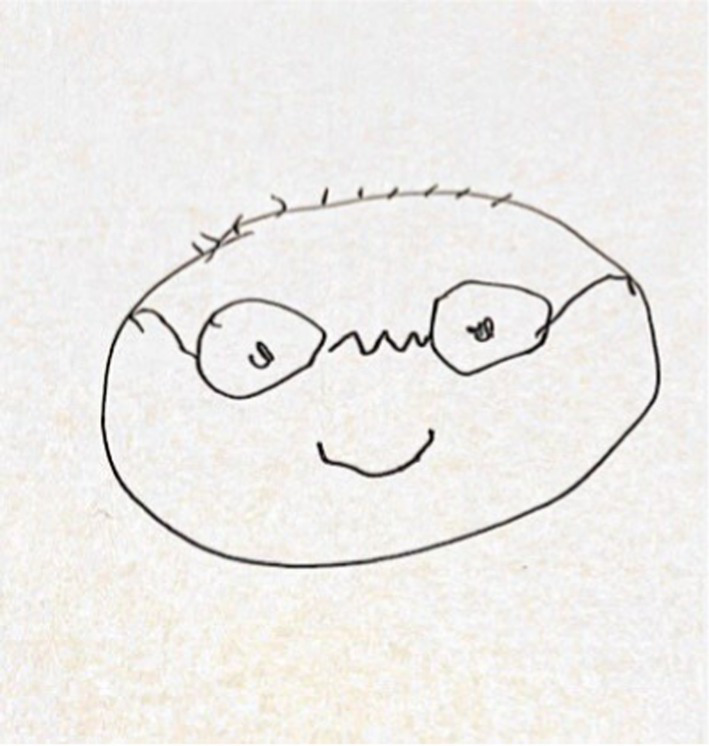
An example of schematic face drawing.

The sixth group (*n* = 56, see Figure 6), labeled ‘Schematic Face Portraits,’ was composed of drawings with schematic and less developed faces, lacking individuality, with a generic facial expression.

**Figure 7:**
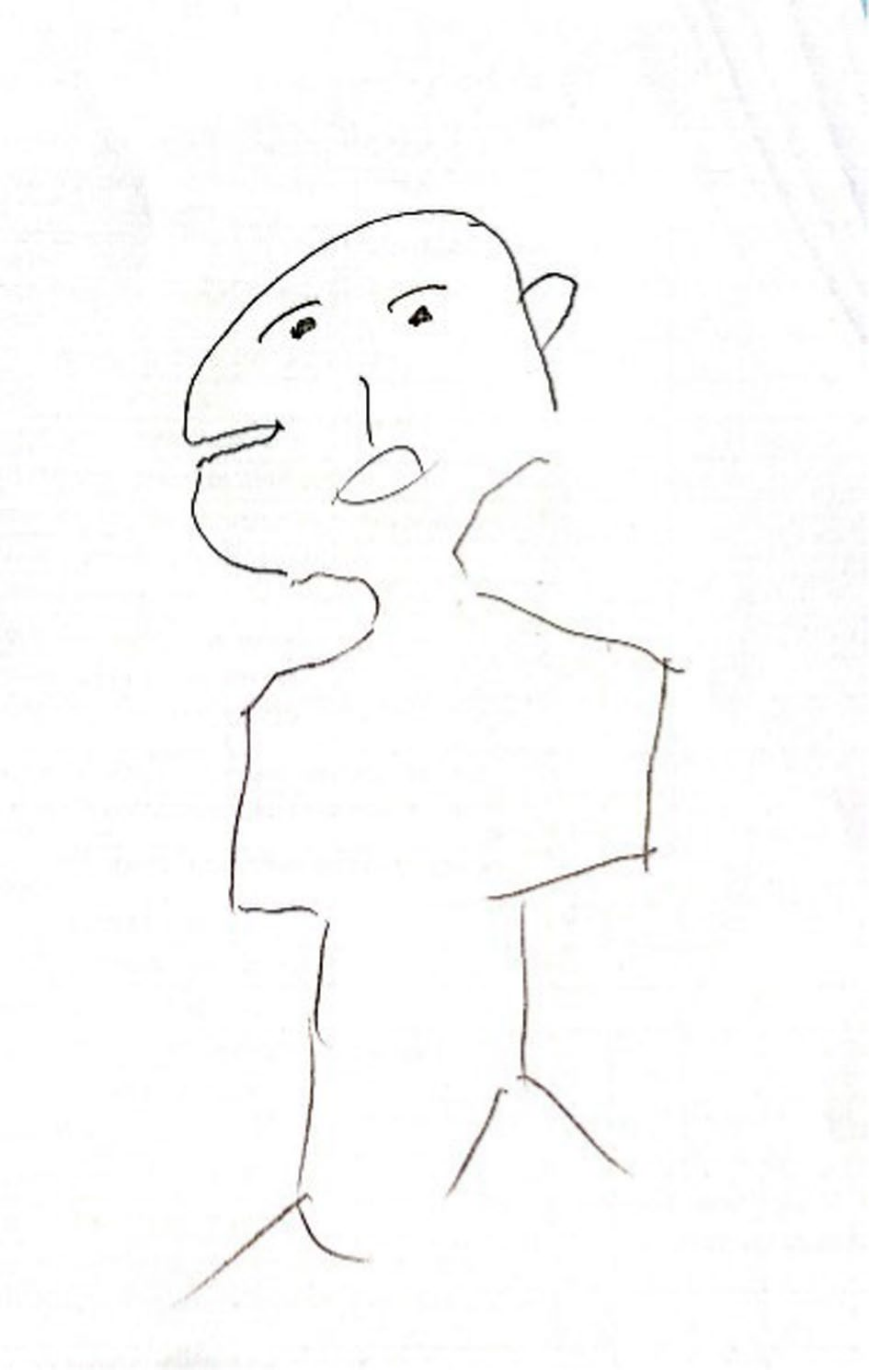
An example of strange drawing.

The seventh group (*n* = 151, see Figure 7) was labeled ‘Strange Portraits.’ It included drawings characterized by odd faces and bodies that deviated from the norm, that were occasionally faceless, occasionally with multiple figures, and a childish appearance or disjointed body parts.

**Figure 8:**
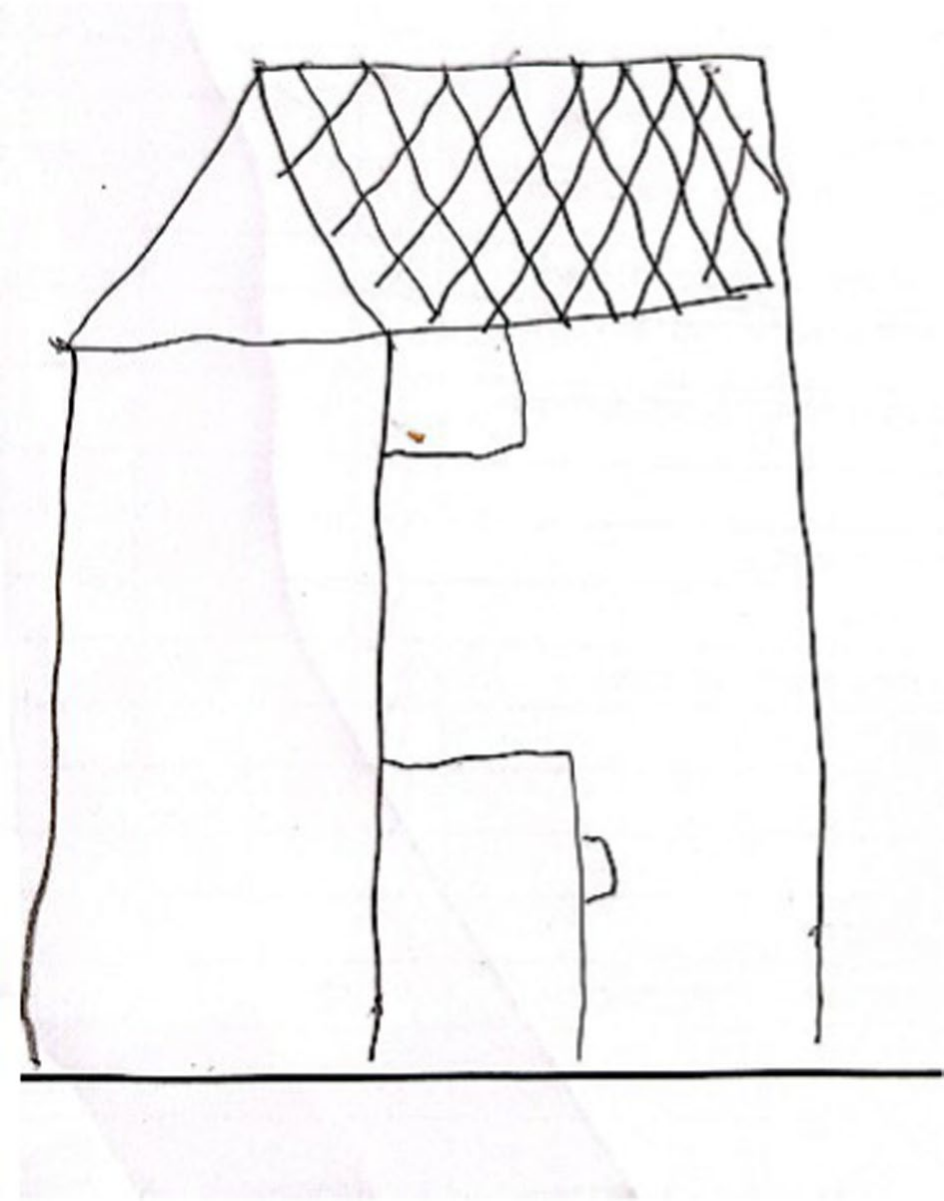
An example of another object drawing.

The eighth group (*n* = 25, see Figure 8) labeled ‘another object’ was composed of drawings of another object rather than a figure.

Inter-rater reliability based on 125 (25.2%) cases between the first two authors was Kappa = 0.88, *p* < 001, 95%CI [0.81, 0.95], Chi-square (49) = 696.29, *p* < 0.001.

#### The Montreal cognitive assessment

The MoCa-5 is a 5-min paper-based test designed to detect cognitive impairment in older patients with symptomatology. It tests attention, verbal learning and memory, executive functions/language, and orientation.^1^ The attention domain is evaluated by the immediate recall of five words, with scores ranging from 0 to 5 (1 point for each word correctly recalled). The executive functions/language domain is assessed using a 1-min verbal fluency test, rated on a scale of 0–9. Orientation is assessed by six items on data and geographic orientation, each worth 1 point for a correct answer. Memory is tested by delayed recall and the repetition of the five words administered on the first task (immediate recall), with scores ranging from 0 to 10. The total scores on the MoCA 5-min range from 0 to 30, and a cutoff score of 24 has demonstrated very good specificity (by correctly identifying 87% of all the healthy participants) and excellent sensitivity when differentiating MCI (90%) and Alzheimer’s disease (AD) (100%) from healthy comparisons. One study reported a high correlation between the MoCA 5-min protocol and the MoCA (*r* = 0.87) (Wong et al., 2015). The test has been validated in various countries and was found to be superior to others, such as the Mini-Mental State Examination (MMSE), in discriminating between individuals with mild cognitive impairment and no cognitive impairment (Andersson et al., 2021; O’Driscoll and Shaikh, 2017; Pinto et al., 2019). The Hebrew version of the MoCa was found to differentiate between cognitively asymptomatic older individuals and those with MCI, with a sensitivity of 94.6% and a specificity of 76.3% (Lifshitz et al., 2012).

### Data analysis

Preliminary analyses employed a t-test to investigate the relationship between participants’ cultural backgrounds (Israeli vs. Thai) and the MoCa-5 scores. Additionally, the relationship between participants’ cultures and drawing groups was examined using a Chi-square. To examine the relationship between participants’ drawing groups and the MoCa categories as a function of culture, we conducted a Chi-square analysis followed by Post-hoc analyses of the standardized residuals. To examine the differences in participants’ drawing categories according to their continuous MoCa-5 score, we used a MANCOVA analysis, controlling for culture, followed by a Tukey post-hoc and pairwise analyses.

## Results

### Preliminary analysis

We first examined the relationship between participants’ cultures (Israeli vs. Thai) and the MoCa-5 scores using a *t*-test. We also examined the relationship between participants’ cultures (Israeli vs. Thai) and drawing groups using a Chi-square, in which participants’ cultures were the independent variable and drawing groups was the dependent variable. The *t*-test for the MoCA-5 indicated a higher mean score for the Thai group than the Israeli group (*t*(274.93) = −2.35, *M*Thai = 20.82 (*SD* = 4.19) *M*Israeli = 19.64 (*SD* = 6.21), Mean Difference = −1.18, [95%CI = −2.180, −0.18]). The chi-square analysis revealed significant differences in drawing groups as a function of culture [*χ*^2^(7) = 24.24, *p* < 0.001, *η*^2^ = 0.11].

*Post hoc* analyses of the standardized residuals revealed that the distribution across the drawing groups was relatively close to the expected counts for the Israeli participants, with residuals indicating minor deviations. Specifically, there was a slight overrepresentation of participants in Group 4 (‘disjointed face and body portraits’) and a slight underrepresentation in Group 1 (‘adapted face portraits’) and Group 2 (‘adapted body portraits’), as indicated by residuals of −4.9 for each. By contrast, there were more pronounced deviations from the expected counts for the Thai participants. Group 6 (‘exaggerated face portraits’) showed a significant overrepresentation, with a positive residual of 7.1, while Group 5 (‘schematic faces’) had a notable underrepresentation, with a residual of −6.3. Additionally, Group 7 (‘strange portraits’) and Group 8 (‘other object’) were underrepresented, as indicated by negative residuals of −3.0 and −5.7, respectively. These findings suggest that the country of origin played a role in the distribution of drawing groups (see Table 1). Therefore, we controlled for culture throughout the analyses.

**Table 1:** Frequency of drawings groups according to culture.

Group	Israel *n*	%	Thailand *n*	%	Total
1	39	68.4	18	31.6	57
2	49	70.0	21	30.0	70
3	24	70.6	10	29.4	34
4	48	84.2	9	15.8	57
5	44	89.8	5	10.2	49
6	36	64.3	20	35.7	56
7	117	79.1	31	20.9	148
8	25	0	0	0	25
Total	382	100	114	100	496

### Differences in participants’ MoCa-5 scores as a function of self-drawing groups

To examine the differences in participants’ drawing categories according to their MoCa-5 scores, an ANCOVA analysis was conducted, with the drawing categories as the independent variable and the MoCa-5 score as the dependent variable, while controlling for culture. A Tukey *post hoc* test and pairwise analyses then followed this analysis. The analysis yielded a significant main effect for the drawing groups *F*(7, 496) = 17.73, *p* < 0.000, *η^2^* = 0.21 and drawing groups × culture interaction *F*(6, 496) = 2.64 *p* < 0.05, *η^2^* = 0.03.

As shown in Table 2, the Tukey *post hoc* analysis indicated that participants in the ‘strange portraits’ group (Group 7) and those who drew another object instead of a figure (Group 8) had the lowest MoCa-5 scores. These scores were significantly lower than those of the participants in the ‘disjointed face and body portraits’ (group 4) and the ‘schematic faces’ (group 5), who in turn scored lower than participants in the adapted face portraits group (Group 1) or the adapted body portraits group (Group 2). The pairwise comparison for the interaction effect indicated that the Israeli participants in the ‘strange portraits’ (group 7) had a lower MoCa-5 score than their Thai counterparts (‘∆ = −3.38, *p* = 0.001, 95%CI = −5.345, −1.432).

**Table 2:** ANOVA analyses: differences in Moca-5 and CDS scores according to drawing groups.

Group	1 *n* = 57 *M* SD	2 *n* = 70 *M* SD	3 *n* = 34 *M* SD	4 *n* = 57 *M* SD	5 *n* = 49 *M* SD	6 *n* = 56 *M* SD	7 *n* = 148 *M* SD	8 *n* = 25 *M* SD	*F*	μ^2^	Tukey contrast
Moca-5 (*n* = 496)	24.14 4.24	24.22 4.83	22.12 4.26	20.28 4.59	20.10 4.69	18.57 5.17	16.55 5.49	16.28 5.54	17.71**	0.21	1,2 > 4,5 > 7,8

**p < 0.01.

### Differences in participants’ MoCa-5 score categories as a function of self-drawing groups

To examine the relationship between participants’ drawing groups and the MoCa categories as a function of culture, a Chi-square analysis was conducted in which the participants’ MoCa categories (severe to mild cognitive impairment versus normal cognitive functioning) was the independent variable, and the drawing group was the dependent variable. A post-hoc analysis of the standardized residuals followed the analysis. The analysis was conducted for the entire sample and separately for participants from each culture. The analysis for the entire sample revealed differences between the drawing groups and the MoCa categories (high/mild to severe) [*χ*^2^(7) = 131.45, *p* = 0.000, *μ*^2^ = 0.49], suggesting that the response proportions of the eight drawing groups differed significantly across the MoCa categories.

Post-hoc analysis of the standardized residuals revealed significant deviations in the proportions of drawing groups within each MoCa category. Participants with severe to mild cognitive impairment showed a significantly higher proportion of drawings in the “strange portraits” group (group 7) than expected (residual = 31.0) and a lower proportion of drawings in the “adapted face portraits” (group 1) (residual = −17.6). By contrast, the normal cognitive functioning group had a significantly higher proportion of drawings in the ‘adapted body portraits’ group (group 2) (residual = 29.00) and a lower proportion of drawings in the “strange portraits” group (group 7) (residual = −31.0).

Associations were also found for the Israeli group [*χ*^2^(7) = 129.52, *p* = 0.000, *μ*^2^ = 0.56] and the Thai group [*χ*^2^(6) = 22.00 *p* = 0.001, *μ*^2^ = 0.28]. *Post hoc* analysis of the standardized residuals for the Israeli sample revealed significant deviations in the proportions of drawing groups within each MoCA category. Participants with severe to mild cognitive impairment showed a significantly higher proportion of drawings in the ‘Strange Portraits’ group (Group 7) than expected (residual = 28.2) and considerably lower proportions of drawings in the ‘adapted face portraits’ group (Group 1) and the ‘Adapted Body Portraits’ group (Group 2) (residuals = −17.9 and −21.1, respectively). In addition, participants with normal cognitive functioning had a significantly higher proportion of drawings in the ‘Adapted Body Portraits’ group (Group 2) (residual = 21.1) and a lower proportion of drawings in the ‘Strange Portraits’ group (Group 7) (residual = −28.2).

*Post hoc* analysis of the standardized residuals for the Thai sample revealed significant deviations in the proportions of drawing groups within each MoCA category. Participants with severe to mild cognitive impairment had a significantly higher proportion of drawings in Group 6 (‘Exaggerated Face Portraits’) than expected (residual = 4.8), and a significantly lower proportion of drawings in Group 2 (‘Adapted Body Portraits’) (residual = −0.6). Participants with normal cognitive functioning had a considerably higher proportion of drawings in Group 2 (‘Adapted Body Portraits’) than expected (residual = 7.9) and a lower proportion of drawings in Group 6 (residual = −4.8). Note, however, that four cells in the Thai sample had expected counts of less than five, which could have affected the reliability of the Chi-square test (Table 3).

**Table 3:** Moca categories according to drawing groups and culture.

Culture	Moca categories/drawing group	Moca score <24	% in sample (MoCA < 24)	Moca score 24–30	% in sample (MoCA 24–30)	Total (n)
Total sample						
	1	22	38.6	35	61.4	57
	2	20	28.6	50	71.4	70
	3	19	55.9	15	44.1	34
	4	42	73.7	15	26.3	57
	5	37	75.5	12	24.5	49
	6	47	83.9	9	16.1	56
	7	134	90.5	14	9.5	148
	8	24	96.0	1	4.0	25
Total		345	69.6	151	30.4	496
Israel						
	1	9	23.1	30	76.9	39
	2	13	26.5	36	73.5	49
	3	12	50.0	12	50.0	24
	4	35	72.9	13	27.1	48
	5	34	77.3	10	22.7	44
	6	28	77.8	8	22.2	36
	7	109	93.2	8	6.8	117
	8	24	96.0	1	4.0	25
Total		264	69.1	118	30.9	382
Thailand						
	1	13	72.9	5	27.8	18
	2	7	33.3	14	66.7	21
	3	7	70.0	3	30.0	10
	4	7	77.8	2	22.2	9
	5	3	60.0	2	40.0	5
	6	19	95.0	1	5.0	20
	7	25	80.6	6	19.4	31
	8	0	0.0	0	0.0	0
Total		81	71.1	33	28.9	114

## Discussion

The current study explored the characteristics of self-figure drawings in older adults with AD, MCI, and normal cognitive functions. The findings revealed significant associations between drawing characteristics and cognitive status. Specifically, participants who produced detailed and adapted drawings categorized as ‘Adapted Face Portraits’ and ‘Adapted Full Body Portraits’ generally scored higher on the MoCA-5, indicating better cognitive functioning. By contrast, individuals with lower MoCA-5 scores reflecting moderate to severe cognitive impairment produced more schematic or distorted drawings that fell into the categories labeled as ‘Schematic Body Portraits,’ ‘Schematic Face Portraits,’ and ‘Strange Portraits.’ These findings support the hypothesis that more detailed and better-proportioned drawings indicate better cognitive functioning, while more superficial, disorganized, or strange drawings are associated with cognitive decline. This is consistent with earlier studies suggesting that drawing characteristics can reflect cognitive abilities and emotional state (Ericsson, 1996; Lev-Wiesel and Hirshenzon-Segev, 2003; Stanzani Maserati et al., 2018).

Drawing is a complex cognitive task that requires the coordination of multiple cognitive functions, including perception, memory, executive function, and motor skills. Individuals with preserved cognitive abilities can produce more accurate and detailed representations of themselves, reflecting the intactness of these cognitive domains (Cicalò, 2017). Here, the participants who exhibited higher cognitive scores were able to draw complete and detailed body figures or face portraits, suggesting that their ability to accurately represent themselves visually was linked to preserved cognitive functions. By contrast, individuals with cognitive impairments, particularly those with AD and MCI, struggled with tasks that required executive functions and visuospatial abilities, which are essential for creating detailed and well-proportioned self-figure drawings (Crutch and Rossor, 2006; Paganini-Hill and Clark, 2007). This difficulty may echo the tendency of individuals with more severe cognitive impairments to produce less detailed, more schematic drawings, which could mirror the erosion of cognitive abilities that underlie the drawing process.

In addition, spatial perception plays a crucial role in producing self-figure drawings. Neuroimaging studies have shown that age-related changes in brain structure, particularly in areas associated with spatial processing, are linked to declines in spatial abilities (Fjell et al., 2013). The changes in spatial perception often associated with aging and neurodegenerative diseases affect individuals’ ability to represent their environment and themselves accurately. Ericsson (1996), for example, noted changes in the structural characteristics of human figure drawings in individuals with dementia. The current findings further support this supposition by revealing differences in drawing coherency, elaboration, and organization that may be indicative of the deficits in spatial perception and visuospatial abilities commonly observed in individuals with AD and MCI (Cronin-Golomb and Amick, 2001).

Self-figure drawings may reflect emotional aspects of the self, particularly in old age. Cognitive decline, emotional factors, and psychological well-being can influence changes in self-concept (Clare et al., 2020; Stites et al., 2018). Some studies suggest that individuals with mild cognitive impairment (MCI) and Alzheimer’s disease (AD) may be more vulnerable to depression (Stites et al., 2018), which has been linked to further cognitive decline (Ismail et al., 2017; Jungwirth et al., 2009). In this study, participants with more severe cognitive impairment and lower MoCA-5 scores tended to produce fragmented or overly simplistic drawings, possibly reflecting both cognitive and emotional factors. The relationship between cognitive decline and emotional distress is complex and may be bidirectional: cognitive impairment can weaken self-concept, while a diminished sense of self might contribute to further cognitive decline (Jetten et al., 2010). This aligns with.

Klein and Gangi’s (2010) “erosion of the self,” where cognitive decline corresponds with a weakening of self-knowledge, often reflected in simplified self-representations. Some research suggests that individuals with cognitive impairments produce drawings with less detail and coherence (Addis and Tippett, 2004). In this study, participants with moderate to severe cognitive impairment tended to create more schematic and fragmented self-figures, while those with higher cognitive scores and more detailed self-portraits may have retained a stronger sense of self. However, further research is needed to clarify these relationships and contributing factors.

Finally, cultural variations in drawing practices must be considered, as demonstrated by the differences observed between the Israeli and Thai participants, with Thai participants having higher MoCA-5 scores than their Israeli counterparts and fewer differences in drawing group distribution. The finding regarding the higher MoCa score contrasts prior findings, which indicated a higher prevalence of dementia in Thailand and in low- and middle-income countries compared to Western countries (Jitapunkul et al., 2001; Kongpakwattana et al., 2019). One potential explanation may be related to the cultural emphasis on precision and conformity in many Asian societies and Thai participants’ focus on memorizing, meeting expectations, and performing tasks with accuracy, leading to better scores on structured cognitive assessments like the MoCA in contrast to Israel’s culture that encourages more open-ended thinking and creative problem-solving, which may not align as closely with the structured nature of the MoCA (Maor et al., 2024).

Furthermore, cultural differences in artistic expression and interpretation likely influence how individuals approach and complete drawing tasks, which should be considered when utilizing self-figure drawings in cognitive assessments (Goodenough, 1926; Koppitz, 1968, 1984). For example, a comparison of self-figure drawings between Thais and Israelis revealed differences in body size and shape, as well as the inclusion of religious and cultural symbols (Binson et al., 2023). Additionally, a study on self-expression through drawings found that Euro-Canadians depicted a larger “self” and were more likely to include a face compared to Chinese participants (Yap et al., 2022).

In the current study, fewer differences in drawing group distribution and a higher rate of schematic drawings were observed among Thai participants. This reduced variation and focus can be attributed to Thai individuals’ emphasis on creating harmonious and pleasing compositions, along with their focus on closed-ended or “how-to-do” exercises and conformity, which align with Thai cultural values prioritizing balance and aesthetics, leading to less diverse and more standardized artworks (Binson et al., 2023). In contrast, Israeli participants were more likely to use drawings as a means of expressing personal experiences or emotions, often reflecting a stronger focus on individuality and self-expression (Binson et al., 2023).

### Clinical implications

These findings have several implications for clinical practice, particularly when assessing and monitoring cognitive decline in older adults. Self-figure drawings, as a non-invasive and cost-effective tool, could provide clinicians with a complementary way to evaluate cognitive status in individuals with varying degrees of cognitive impairment, including those with AD and MCI. Clinicians working in settings with limited access to comprehensive neuropsychological testing—such as in community health, nursing homes, or non-Western cultural contexts—may find self-figure drawing tasks particularly valuable for screening purposes. The detailed nature of self-representations, as demonstrated in the “Adapted Face Portraits” and “Adapted Full Body Portraits” groups, may indicate preserved cognitive function. More schematic and fragmented drawings could signal the need for further assessment or intervention.

Inspired by previous research (e.g., Goldner and Frid, 2021), our approach to categorizing drawings proved effective in differentiating cognitive states. By examining the organization, impression, and aggregation of signs in the drawings, we captured a broader spectrum of cognitive functions than would have been possible by focusing solely on individual drawing features. This holistic approach, combined with the use of the MoCA-5, provided a more comprehensive assessment of cognitive function, thus enhancing the reliability of the findings.

Given the link between emotional well-being and cognitive function, clinicians should pay attention to the emotional features reflected in self-figure drawings. Emotional distress, such as depression, can worsen cognitive decline and impact self-perception, as evident in the relationship between emotional changes and schematic or distorted drawings. Incorporating assessments of emotional health alongside cognitive evaluations could improve patient care by providing a more comprehensive approach to managing cognitive decline.

Finally, cultural differences in artistic expression and interpretation are likely to influence how individuals engage with and perform drawing tasks. Thus, to optimize the clinical utility of self-figure drawings in cognitive assessments, it is essential to implement culturally sensitive approaches and standardized scoring systems.

### Limitations, future research, and conclusion

The current study has several limitations. First, the correlational design precluded drawing causal conclusions about the relationship between drawing characteristics and cognitive decline. Longitudinal studies could reveal how drawing characteristics evolve over time as cognitive impairment progresses, thereby complementing the cross-sectional designs commonly employed in most research (Bonoti et al., 2015; Crutch and Rossor, 2006; Jungwirth et al., 2009). Additionally, although our sample size was considerably larger than those in previous studies (e.g., Lev-Wiesel and Hirshenzon-Segev, 2003), the unequal proportion of participants from Israel and Thailand may have affected the generalizability of the findings. Moreover, the number of participants in each drawing group was uneven and relatively small. Therefore, the findings should be interpreted with caution. Finally, although we controlled several demographic variables, other factors, such as participants’ artistic abilities and prior experience with drawing, were not considered. These variables could have potentially influenced the quality and characteristics of the drawings and should be considered in future research. Nevertheless, despite these limitations, using self-figure drawings constitutes a promising addition to the cognitive assessment toolkit, offering valuable insights into the cognitive and emotional states of older adults. Future research and clinical trials should continue to explore and refine this approach, considering both cognitive and emotional dimensions when assessing cognitive impairment.

## Data Availability

The raw data supporting the conclusions of this article will be made available by the authors, without undue reservation.

## Footnotes

^1^ https://mocacognition.com/
